# 肺癌侵袭转移相关microRNAs的研究进展

**DOI:** 10.3779/j.issn.1009-3419.2010.03.18

**Published:** 2010-03-20

**Affiliations:** 300052 天津，天津医科大学总医院，天津市肺癌研究所，天津市肺癌转移与肿瘤微环境重点实验室 Tianjin Key Laboratory of Lung Cancer Metastasis and Tumor Microenviroment, Tianjin Lung Cancer Institute, Tianjin Medical Univercity General Hospital, Tianjin 300052, China

肺癌侵袭转移是肺癌的恶性标志和特征，也是肺癌患者治疗失败和死亡的主要原因。肺癌侵袭转移是一个多因素调控、多步骤、多阶段、连续复杂的生物学过程，是癌细胞从原发部位转移到远端部位形成肿瘤的过程^[1, 2]^，主要包括肿瘤细胞脱离原发灶、进入循环系统（血液循环与淋巴循环）、侵袭靶器官、远处克隆和血管生成形成转移灶。肺癌侵袭转移涉及多因素、多水平的调节。MicroRNA（miRNA）是一类高度保守、长度很短的非编码调控单链RNA，约20 nt-24 nt，通过与目标mRNA互补配对导致mRNA降解或抑制转录后翻译诱导基因沉默。miRNA参与生命过程中一系列的重要进程，包括发育、造血、器官形成、凋亡和细胞增殖、癌症的发生和发展等。近年来大量研究^[3, 4]^表明，miRNAs与人类多种肿瘤的发生、发展及侵袭转移存在着密切关系。迄今，有关miRNA与肺癌侵袭转移相关的研究已有较多报道，但其确切机制尚未明确，随着人们对miRNAs研究不断深入，已证明miRNAs在肺癌侵袭转移调控中发挥着重要作用。

## 1 miRNAs调节肿瘤细胞转移的分子机制

### 1.1 调节癌基因或抑癌基因表达miRNAs可通过下调抑癌

基因表达，促进肿瘤细胞侵袭转移。Asangani等^[5]^发现在结肠癌细胞cob206f中*PDCD4*为miR-21的靶基因，二者呈负相关，指出miR-21是通过抑制PDCD4的表达而影响肿瘤细胞浸润转移的。*PDCD4*已被证实为肿瘤抑制基因，PDCD4能与转录起始因子eIF4A和eIF4G相互作用而抑制基因转录。在66%的肺癌患者中，miR-21至少有2倍的上调^[6]^。抑癌基因*PTEN*和*sprouty2*（*SPRY2*）也是miR-21的靶基因。Meng等^[7]^分析了正常肝细胞和肝癌细胞中197种miRNAs的表达，发现肝癌细胞中miR-21的含量显著高于正常肝细胞，揭示了miR-21下调PTEN的表达，进而通过影响PI3K信号途径而促进肿瘤细胞的增殖和转移。Sayed等^[8]^在结肠癌细胞SW480中下调miR-21表达后，发现肿瘤细胞的迁移能力显著下降，并证实了这一过程是通过上调其靶基因*SPRY2*的表达，使细胞微绒毛活动受限而实现的。miRNAs可通过下调癌基因表达而抑制肿瘤细胞的侵袭转移。Li等^[9]^研究表明，在肝癌细胞中miRNA-34a可下调原癌基因*c-MET*的mRNA水平和蛋白水平的表达，从而降低*c-MET*诱导的细胞外信号调节激酶ERK1/2的活性，抑制肿瘤细胞侵袭转移。

### 1.2 调节转移相关基因表达

miRNAs通过调节*SOX4*、*RDX*、*RhoA*等肿瘤转移相关基因表达，调控肿瘤细胞的侵袭转移。在乳腺癌细胞中miR-335通过靶向抑制转录因子SOX4和细胞外基质成分钙粘蛋白C的表达，调控肿瘤细胞的增殖，抑制乳腺癌细胞的侵袭和转移^[10]^。Cash以及Valastyan等^[11, 12]^研究发现，miRNA-31可通过抑制*Fzd3*、*ITGA5*、*MMP16*、*RDX*和*RhoA*等促进转移基因的表达水平，抑制乳腺癌细胞的侵袭转移。Huang等^[13]^将高表达miRNA-373或miRNA-520c的非转移乳腺癌细胞接种小鼠后，在小鼠的骨骼、脑和肺均产生了转移灶，而对照组未发现转移。研究证实，miRNA-373和miRNA-520c主要通过抑制其靶分子透明质烷表面受体CD44蛋白的表达而促进肿瘤细胞侵袭和转移，*CD44*是一种肿瘤转移抑制基因。在前列腺癌细胞中，也发现了miRNA-373和miRNA-520c可通过调节CD44而促进肿瘤细胞的转移^[14]^。

### 1.3 调节肿瘤细胞上皮间质转化

上皮间质转化（epithelial-mesenchymal transition, EMT）是上皮细胞在形态学上发生向成纤维细胞或间充质细胞表型转变，并获得迁移的能力。一些研究^[15-19]^证明EMT是肿瘤侵袭转移的一个重要步骤，有许多miRNAs参与EMT调节。miRNA-200家族成员miRNA-200a、miRNA-200b、miRNA-200c、miRNA-141和miRNA-429是EMT的重要调节因子^[20]^。miR-200家族可靶向作用于E-cadherin抑制因子ZEB1、ZEB2、SIP1和转录因子8，从而抑制上皮间质的转化^[21-23]^。还有研究^[24]^发现ZEB1与miR-141及miR-200c之间存在着互反馈回路，即miRNA可下调ZEB1的表达，反过来ZEB1还可下调miR-141和miR-200c的表达，这一回路受到环境因子的影响，通过调控EMT过程参与肿瘤细胞的侵袭转移。

### 1.4 调节肿瘤细胞血管生成

肿瘤血管生成是指肿瘤细胞通过分泌促血管生成因子激活静息状态的内皮细胞，使之增殖、迁移至肿瘤附近，并形成毛细血管网包绕肿瘤。肿瘤的生长有赖于新生血管提供血液灌注，为肿瘤组织提供氧气和营养。新生的肿瘤血管管壁薄弱，基膜不完整，通透性高，是肿瘤组织发生转移的结构基础。Sufu和Fus-1在肿瘤转移中具有抑制功能，microRNA-378可靶向抑制Sufu和Fus-1的表达，促进肿瘤细胞生长与肿瘤血管生成，加速肿瘤转移^[25]^。miR-130a可下调GAX（growth arrest-specific homeobox）和HOXA5（homeobox protein hox A5）的表达，Chen以及Rhoads等^[26, 27]^通过反义寡核苷酸技术降低miR-130a表达后，HOXA5蛋白过表达，进而下调VEGFR、HIF-1等促血管生成因子的表达，抑制肿瘤转移。

### 1.5 调节肿瘤细胞DNA甲基化模式

miRNAs可通过抑制DNA甲基化相关酶，改变肿瘤细胞的DNA甲基化状态，从而调控肿瘤的侵袭转移。Fabbri等^[28]^通过实验证明，在肺癌细胞A549和H1299中miR-29家族通过下调甲基转移酶3A（DNMT3A）和甲基转移酶3B（DNMT3B）的表达，减少肿瘤抑制基因*FHIT*和*WWOX*启动子甲基化，抑制肿瘤形成。Duursma等^[29]^研究发现，在乳腺癌细胞MCF-7中miR-148可抑制DNMT3B表达。同时，一些肿瘤转移相关miRNAs也受到甲基化调节。有研究发现miR-148a、miR-9家族的3个成员和miR-34b/c簇为超甲基化miRNA，它们呈现肿瘤特异性甲基化，这些miRNA的沉默可介导致癌基因和转移相关基因的活化，如miR-34b/c可促使E3F3、C-MYC和CDK6激活，而miR-148a可促进TGIF2活化。在人原发性肿瘤中，miR-34b/c的甲基化与癌基因的上调明显相关，可见在体内这些癌基因也是miRNA作用的靶标，而且miRNA的表观遗传学沉默可导致其在肿瘤患者体内高表达^[30]^。

### 1.6 调节肿瘤细胞粘附力

miRNAs通过调节肿瘤细胞粘附分子，改变细胞粘附力与侵袭力，实现调控肿瘤细胞的侵袭转移。在黑色素瘤细胞中，let-7a可下调integrin β3的表达水平，同时抑制了肿瘤细胞的侵袭^[31]^。CD44也是介导细胞与细胞外基质之间粘附的重要分子，在乳腺癌和前列腺癌细胞中均发现miRNA-373和miRNA-520c可抑制其靶基因*CD44*的表达，从而促进肿瘤细胞侵袭和转移^[13, 14]^。有研究发现，E-钙粘蛋白为miR-9的靶基因，miR-9在肝癌细胞中高表达，可降低E-钙粘蛋白的表达水平，抑制肿瘤细胞的转移^[32]^。

## 2 肺癌侵袭转移中miRNAs的调节功能

### 2.1 let-7

let-7是较早发现的与肺癌细胞侵袭转移相关的miRNA家族，也是目前研究最为详细的microRNA之一。目前已发现的pre-let-7家族成员多达125种，人类含有11种pre-let-7（hsa-let-7a-1, 2, 3、hsa-let-7b、hsa-let-7c、hsa-let-7d、hsa-let-7e、hsa-let-7f-1, 2、hsa-let-7g、hsa-let-7i），共产生8种Mat-let-7，它们彼此基因序列高度保守，功能相似。let-7可以调控*Hmga2*基因的表达，HMGA2蛋白是一种高迁移率组A蛋白，在90%以上的肺癌中都有表达，而且与肺癌患者的存活率呈负相关，let-7通过HMGA2这一靶基因而调控肺癌细胞的迁移能力^[33]^。抑制let-7在正常NIH3T3细胞中的表达，可以诱导细胞恶性转化^[34]^。let-7通过多种途径抑制肿瘤细胞增殖。Johnson等^[35]^首先发现Ras家族蛋白也是let-7的靶蛋白，Ras的3'UTR含有多个与let-7互补的位点，let-7低表达可上调Ras蛋白表达水平，进而通过Ras蛋白这个膜相关GTPase信号蛋白调节细胞生长和分化。在K-ras突变的非小细胞肺癌（non-small cell lung cancer, NSCLC）细胞A549和Calu-1中诱导表达let-7g，发现可几乎完全抑制肿瘤在体内的形成，而未突变的NSCLC细胞H1650则只部分抑制了肿瘤的形成。重新过表达外源K-RasG12D又能拮抗let-7g引起的肿瘤抑制，而重新表达外源HMGA2则只能有限的影响let-7g抑制肿瘤的作用。let-7在肿瘤细胞中的过表达还可以改变细胞的细胞周期进行，降低肿瘤细胞分裂并诱导肿瘤凋亡^[36, 37]^。

### 2.2 miR-200

在NSCLC细胞系A549中，Hurteau等^[21]^通过上调miR-200c的表达水平，发现带锌指结构的转录因子8（TCF8, ZEB1）的作用缺失，进而抑制了E钙黏蛋白的表达，导致细胞形态改变，黏附力下降。E黏蛋白是EMT重要的诱导物，表明miR-200c通过调节EMT来抑制肺癌细胞的侵袭转移。

### 2.3 miR-183

Wang等^[38]^研究发现，miR-183可以抑制肺癌细胞的迁移和侵袭能力。他们研究发现miR-183的表达水平与肺癌细胞的转移能力呈负相关。在肺癌细胞中将miR-183过表达，能够抑制肺癌细胞的迁移和侵袭。进一步研究其机制发现，VIL2编码的蛋白Ezrin是miR-183的作用靶点，同时还发现miR-183可以调节其他与迁移和侵袭相关基因的表达水平，miR-183可能为一种肿瘤转移抑制因子。

### 2.4 miR-125a-5p

Wang等^[39]^近期研究发现，miR-125a-5p可以抑制肺癌细胞的侵袭转移能力。EGFR（epidermal growth factor receptor, EGFR）信号转导途径和microRNA在肺癌的发生和发展中均起着重要的作用，为了研究miRNA在EGFR信号转导途径中的作用，他们应用miRNA芯片检测发现miR-125a-5p为EGFR信号途径的下游作用因子。进一步研究发现，miR-125a-5p能够抑制肺癌细胞的迁移和侵袭。此外，miR-125a-5p还可调节EGFR信号通路中多个下游基因的表达。检测肺癌样本中miR-125a-5p的表达水平，结果显示miR-125a-5p抑制和肺癌的发生显著相关。证明了miR-125a-5p是一个受EGFR信号途径调节的肿瘤转移抑制因子。

### 2.5 miR-126

Crawford等^[40]^研究发现，在肺癌细胞中Crk蛋白可能为miR-126的靶蛋白。Crk可通过参与调节受体酪氨酸激酶和整合蛋白等多种分子的信号转导通路，而改变细胞的粘附、增殖和迁移能力，增加Crk的表达可以促进肿瘤细胞的侵袭性。他们在肺癌细胞系中过表达miR-126后，抑制了Crk的蛋白表达水平，但对其mRNA表达水平无影响。过表达miR-126的肺癌细胞表现出粘附、迁移和侵袭能力的下降。证明了miR-126通过部分调节Crk抑制肺癌细胞的粘附、迁移和侵袭表型。

### 2.6 miR-221 & 222

Garofalo等^[41]^发现，在TNF相关凋亡诱导配体（TNF-related apoptosis-inducing ligand, TRAIL）抵抗的非小细胞肺癌细胞中，miR-221和miR-222表达水平升高。最近的研究^[42]^发现，在非小细胞肺癌和肝癌细胞中，miR-221 & 222在侵袭性肿瘤细胞的表达水平显著高于低侵袭性肿瘤细胞和/或正常细胞中的表达水平。进一步研究发现MiR-221 & 222通过作用其靶基因肿瘤抑制因子PTEN和TIMP3，诱导TRAIL抵抗，并通过激活AKT通路和金属蛋白酶促进细胞迁移。还发现癌基因*MET*可通过调控c-Jun转录因子激活miR-221 & 222的表达。

### 2.7 miR-17-92

miR-17-92家族在肺癌尤其是小细胞肺癌中高表达，在肺癌发生发展中起到原癌基因的作用。将miR-17-92在A549细胞中过表达后，细胞的增殖能力显著提高，而将它的成员如miR-18a、miR-19a、miR-20a单独转染至A549中并不能促进细胞增殖，表明miR-17-92家族作为一个整体发挥作用^[43]^。目前，对于miR-17-92家族在肺癌细胞中的作用机制尚不完全清楚。Lewis等^[44]^推测抑癌基因*PTEN*和*RB2*可能为miR-17-92家族的靶基因，其塔肿瘤研究中发现miR-17-92家族能与癌基因*Myc*协同作用^[45]^。已有研究^[46]^证实，p-Akt的过表达和PTEN的低表达与非小细胞肺癌的迁移和侵袭密切相关，提示miR-17-92可能通过调控PTEN的表达促进肺癌细胞的侵袭和转移。

### 2.8 miR-1

Nasser等^[47]^研究发现micro-RNA-1（miR-1）在正常肺组织中表达，而在人原发肺癌组织和肺癌细胞中低水平表达。原位杂交实验结果显示miR-1定位于支气管上皮细胞，在肺癌细胞中，肿瘤抑制因子C/EBPα和组蛋白脱乙酰酶抑制剂可以重新活化的miR-1的表达，在肺癌细胞中miR-1表现为抑癌基因的作用。在不表达miR-1的A549和H1299细胞中过表达miR-1后，将稳定转染的肺癌细胞接种裸鼠，成瘤实验显现miR-1过表达后，A549和H1299细胞的成瘤性、肿瘤生长和转移性均受到了显著的抑制。同时研究还发现，miR-1过表达可显著下调原癌基因*MET*、*Pim-1*、*FoxP1*和*HDAC4*的表达水平。

## 3 结语

阐明miRNAs的功能和作用机制，有助于在转录后水平上深入研究基因表达调控，而且有助于从新的角度和层面诠释人类疾病发病机制，并由此寻求新的、更有效的诊治方法。已有的研究结果表明miRNA作用机制的复杂性，如一个miRNA可以同时调控多个靶基因，一个靶基因也可以同时受到多个miRNA的调控，同时miRNA可以受到一些细胞因子及甲基化等表观遗传学机制的调控等。迄今为止，仍有许多与肿瘤转移相关的miRNAs未被发现，许多miRNAs的功能及其在肿瘤侵袭转移中的机制还有待研究。在临床上，如何应用miRNAs调控转移表型理论与模式，对肿瘤转移进行早期诊断和治疗仍存在许多未知有待深入探究。
